# EGFP标记的人肺癌裸鼠原位移植模型的建立

**DOI:** 10.3779/j.issn.1009-3419.2010.07.03

**Published:** 2010-07-20

**Authors:** 淑珍 魏, 宇 孙, 志坚 杨, 勇 宋

**Affiliations:** 1 210002 南京，南京大学医学院 Medical College, Nanjing University, Nanjing 210093, China; 2 210002 南京，南京大学临床学院南京军区南京总医院 Department of Respiratory Medicine, Nanjing University School of Medicine, Jinling Hospital, Nanjing 210002, China; 3 210002 南京，南京源端生物科技有限公司 Nanjing Origin Center for Tumor Animal Model, Nanjing 210002, China

**Keywords:** 肺肿瘤, 增强型绿色荧光蛋白, 外科原位移植, 模型, Lung neoplasms, Enhanced green fluorescent protein, Surgical orthotopic implantation, Model

## Abstract

**背景与目的:**

小鼠活体分子成像模型可以连续实时监测活体肿瘤的变化。本研究拟通过外科原位移植法建立表达绿色荧光蛋白的肺癌裸鼠原位移植模型并探讨其肿瘤生物学特性，从而建立一个良好的肺癌动物实验研究平台。

**方法:**

利用逆转录病毒转染法将增强型绿色荧光蛋白基因导入人肺癌大细胞系NCI-H460，采用外科原位移植法建立肺癌原位移植模型。定期通过小动物活体荧光成像系统观察肿瘤生长，利用相关性检验分析荧光面积和肿瘤体积之间的相关关系，并观察原位移植术后裸鼠的生存期和肿瘤转移情况。

**结果:**

模型建立后1周通过皮瓣在荧光体视镜下可观察到肺部肿瘤的绿色荧光，成瘤率为100%。荷瘤裸小鼠平均生存期为34.2天。解剖裸鼠观察到肿瘤侵及对侧肺、纵隔及肺门淋巴结、胸膜和膈肌，转移率分别为87.5%、75%、25%和12.5%。肿瘤体积和荧光面积具有相关性（*r*=0.873, *P*=0.001）。

**结论:**

外科原位移植法建立的表达EGFP的裸鼠肺癌原位模型是肺癌临床前研究的理想的实验工具。应用小动物活体荧光成像系统能够定量客观评价肿瘤在动物体内的生长、侵袭和转移，该模型可应用于肺癌的基础研究和新药开发。

## Abstract (en)

**Background and objective:**

*In vivo* molecular imaging with mouse model could continuously and in real-time monitor the changes of the tumor. The aim of this study is to establish stable enhanced green fluorescent protein (EGFP) expressing NCI-H460 cell lines and relevant mouse model via orthotopic transplantation, and to study the characteristic of this model and the quantitative detection method of the primary tumor and metastatic lesions.

**Methods:**

Human lung cancer NCI-H460 cells were transfected with retroviral vector containing the EGFP. Stable high-level expression of EGFP was maintained in the subcutaneously-growing tumors. Fragments of the subcutaneously growing tumor, which were comprised of EGFP-expressing cells, were implanted by surgical orthotopic implantation (SOI) in the lung of nude mice. The dynamic growth of orthotopic tumor was observed using *in vivo* fluorescence imaging. The correlation of fluorescence area and tumor volume was tested.

**Results:**

After the model established, green fluorescent can be observed through the flap in day 7. Tumor formation rate was 100%. Mean survival time of tumor-bearing nude mice was 34.2 days. The metastasis sites were the contralateral lung, mediastinal and hilar lymph nodes, pleura and diaphragm; metastasis rates were 87.5%, 75%, 25% and 12.5%, respectively. Tumor volume and fluorescence area was correlated (*r*=0.873, *P*=0.001).

**Conclusion:**

The nude mouse model bearing orthotopic human lung cancer expressing EGFP has been successfully established. The model might be used for further molecular studies on tumor metastasis, angiogenesis and also be applied to anti-tumor drug screening in preclinical stage.

肺癌已成为全球癌症死亡的首要原因。即使积极行局部和全身治疗，多数患者仍死于肺癌转移。因此这一现状向肺癌基础研究提出了挑战，需要研发更多更新的治疗方法来提高诊断和治愈率。肺癌动物模型的发展可能会帮助理解肺癌的肿瘤生物学特性，促进早期诊断方法和新的治疗方法的研究。动物肺癌移植瘤模型分为皮下移植模型和原位移植模型^[1]^。目前用于研究的模型多是皮下移植模型，但皮下移植的肿瘤属于异位种植，脱离了其起源组织器官的微环境。许多肿瘤的转移表型是在原位种植后才能表达，因此应用此模型得到的实验结果与临床应用的实际效果之间常有较大的差异。原位种植肺癌动物模型应用肿瘤细胞悬浮液行气管内、胸内、静脉注射，或者将新鲜肿瘤组织块手术原位种植至免疫缺陷动物呼吸系统的组织内。该模型可提高成瘤率，具有较强的侵袭及转移特性^[2]^。相关实验还证明，在肺癌原位移植模型中，瘤块外科原位移植法（surgical orthotopic implantation, SOI）^[3]^比肿瘤细胞悬液原位注射法（cellular orthotopic injection, COI）^[4]^转移率高，其转移的发生具有与临床相似的时间依赖性。

近年来，荧光蛋白的深入研究及原位动物模型的广泛应用使在体肿瘤生长和转移的非侵入性实时成像成为现实。将荧光蛋白基因转染至肿瘤细胞，追踪体内肿瘤细胞中荧光蛋白的表达，可实时监测原发肿瘤的生长和转移，在细胞和分子水平对活体内的生理和病理过程进行定性或定量可视化观察。活体荧光显像技术为直接观察肿瘤的发生、生长、转移、血管生成和肿瘤细胞与宿主微环境的相互作用等生物学行为及直接客观评价抗肿瘤药物疗效提供了重要的手段。目前国内该技术在肺癌方面的研究报道也仅限于皮下模型^[5]^和COI^[6]^，而SOI法建立的肺癌模型未见报道。因此本研究采用SOI法将稳定、高表达EGFP的人肺癌细胞系NCI-H460原位移植，建立肺癌裸鼠原位移植模型，并应用小动物活体荧光成像系统观察肿瘤的生长，探讨其生物学特性，以期建立一个理想的动物模型用于研究肺癌的转移和临床前期药物研发。

## 1 材料与方法

### 1.1 材料

#### 1.1.1 细胞系

人非小细胞肺癌NCI-H460：购自美国ATCC（American Type Culture Collection美国组织细胞库）。

#### 1.1.2 EGFP表达载体

PT67包装细胞内含新霉素耐药基因的pLEIN-EGFP逆转录病毒，由美国Anticancer公司Hoffman博士惠赠。

#### 1.1.3 实验动物

BALB/c（nu/nu）裸鼠，雌雄不限。4周龄-6周龄，22只，体重18 g-22 g。所有裸鼠在SPF级屏障系统中饲养并进行实验（实验室使用许可证编号：SYXK（苏）2007-0011）。饲养室相对湿度为（55±10）%，温度为（22±2）℃，光照12 h明暗交替，裸鼠饲养用的饲料为钴^60^辐射灭菌过的大小鼠专用颗粒饲料（江苏省协同医药生物工程有限公司）。

### 1.2 方法

#### 1.2.1 表达绿色荧光蛋白的H460肺癌细胞系的建立

在含10%胎牛血清、青霉素、链霉素的RPM1-1640上清液中培养PT67包装细胞。当细胞达到80%-90%融合浓度时收集培养液，经孔径为0.45 μm的醋酸纤维滤膜过滤后备用。转染前24 h更新NCI-H460新鲜培养基，加入含逆转录病毒液的培养基转染4 h后，置于37 ℃孵育箱孵育36 h，在荧光显微镜下观察荧光。收集转染细胞，在含200 μg/mL的G418选择性培养基培养细胞。逐步将G418浓度增加200 μg/mL-400 μg/mL，直至抗性克隆形成。用96孔板单个细胞分离方法分离高表达GFP的克隆株，混合分离克隆株，使用常规方法扩增。经体外连续传代培养3个月，检测荧光的表达强度。

#### 1.2.2 原位移植模型的建立

将H460-GFP细胞悬浮于PBS液中，调整细胞浓度为5×10^6^/mL。消毒裸鼠左侧腋下皮肤，将0.2 mL细胞悬液用1 mL带有27G1/2针头的注射器接种于2只裸鼠皮下，形成一皮丘，干棉签压迫。当皮下移植瘤长至直径约10 mm时剥取移植瘤，选择生长良好、瘤结无破溃的荷瘤裸鼠，在净化工作台内，无菌操作下完整剥离瘤结，生理盐水洗去血渍。剪开瘤结，清除中心的坏死组织，剪成直径约1 mm大小的米粒样小瘤块。20只裸小鼠接受外科原位移植术。异氟烷吸入麻醉，将裸鼠置于右侧卧位，四肢适当固定。消毒左侧胸壁皮肤，在近第4、5肋间位置的皮肤切一0.4 cm-0.5 cm的横切口，锐性分离胸壁肌肉，暴露肋骨和肋间肌，打开胸腔。用镊子固定左肺，将准备好的瘤块缝合在左肺上，无菌缝合线缝合关闭胸腔。立即检查缝合情况，如果漏气，继续缝合，直至胸壁完全缝合。用5 mL注射器做胸腔内穿刺抽气，缝合胸壁肌肉和皮肤。裸鼠放回原饲养笼，继续饲养。

#### 1.2.3 监测指标

术后每天观察裸鼠有无并发症及异常情况（活动，神经功能，呼吸，动物外观）。肿瘤种植后每周麻醉处死2只裸鼠，共4周。锐性分离左侧皮肤肌肉。在荧光体视显微镜下打开胸腔和腹腔，分别在自然光源和荧光激发状态下拍照。使用小动物活体成像系统检测GFP的表达，发射波长为520 nm，激发波长为480 nm，曝光时间为1 s，IPP软件定量分析荧光面积。观察原位移植瘤生长情况，经典方法测量肿瘤体积：用游标卡尺测量肿瘤的最长径（a）和与之垂直的最短径（b），根据公式计算肿瘤体积：肿瘤体积（V）=ab^2^/2（a、b以mm为单位）。以肿瘤接种天数为横坐标，肿瘤接种部位荧光面积为纵坐标，绘制原位肿瘤生长曲线。

其余裸鼠每周打开胸部皮瓣，戊巴比妥钠45 mg/kg腹腔注射麻醉裸鼠，或者用手固定裸鼠，在自然光源和荧光激发状态下拍照。当裸鼠出现恶病质后，解剖荷瘤鼠，肉眼观察肿瘤生长情况及周围脏器受累情况，并拍照。

### 1.3 统计学处理

采用SPSS 13.0软件进行统计分析，荧光面积和肿瘤体积用Mean±SD表示，*Pearson*相关性检验分析活体肿瘤面积与肿瘤体积之间的相关性，以*P* < 0.05为差异具有统计学意义。

## 2 结果

### 2.1 EGFP标记的NCI-H460细胞的生物学特性

EGFP标记的NCI-H460细胞为多边形上皮样细胞，贴壁生长，荧光显微镜观察可见细胞表达的荧光信号较强，转染率接近100%，细胞在体外能够稳定表达绿色荧光蛋白，并且随体外长期传代培养无明显消退（图 1）。

**1:**
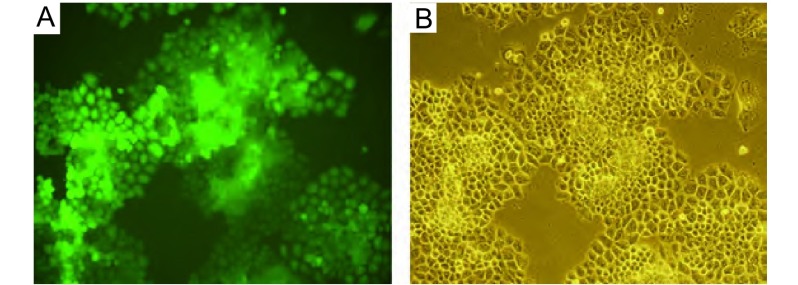
稳定表达EGFP的H460细胞单克隆（×100）。A：荧光显微镜；B：倒置显微镜。 Stable and high-level of EGFP expression in H460 cells *in vitro*. A: Image of NCI-H460-EGFP cells visualized with an inverted fluorescence microscopy; B: Image of (A) visualized with an inverted microscopy.

### 2.2 活体荧光成像动态观察裸鼠原位移植瘤的生长

接受外科原位移植术的裸鼠，术中死亡4只，安全度过手术期的16只裸鼠均完成实验。肺癌原位移植后成瘤率为100%。第7天可活体观察到肺癌原位移植瘤荧光，第3周左右出现转移灶，第28天左侧胸腔被肿瘤占据（图 2）。应用小动物活体荧光成像系统和胸部皮瓣可连续监测绿色荧光蛋白在裸鼠肺部的表达，随着肿瘤移植时间的增加，表达绿色荧光蛋白面积逐渐增大（图 3）。用于观察生存期的8只裸鼠在30天-35天自然死亡，平均生存期为34.2天，称取裸鼠原位移植瘤质量，平均瘤重为（0.66 ±0.25）g，最大者为1.11 g，最小者为0.52 g。处死裸鼠后，行大体观察及荧光显微镜下观察，可见不同程度的转移。与光学成像相比，荧光显像对小的转移灶显像较为清晰（图 4）。7只裸鼠出现右肺转移，6只出现纵隔、肺门淋巴结转移，2只出现胸腔积液，1只出现膈肌转移，而肝、肾、肾上腺、骨骼未见肿瘤浸润。对侧肺、纵隔及肺门淋巴结、胸膜和膈肌的转移率分别为87.5%、75%、25%和12.5%。

**2:**
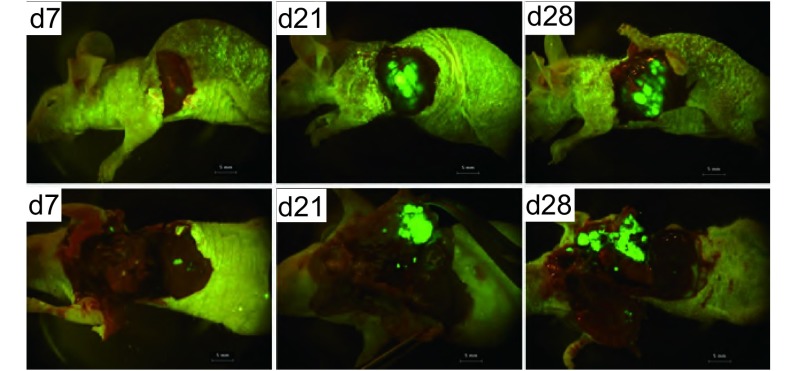
裸鼠原位种植瘤的活体荧光成像动态观察（Bar, 5 mm）。肺癌原位移植后成瘤率为100%，第7天可活体观察到肺癌原位移植瘤的荧光表达，第21天左右出现转移灶，第28天左右裸鼠开始出现消瘦、活动减退、呼吸短促等症状，肿瘤布满整个左侧胸腔。 Dynamic observation of fluorescence imaging in nude mice bearing tumor (Bar, 5 mm). Tumor formation rate was 100% after surgical orthotopic implantation. Green fluorescence in situ can be observed at day 7. Metastases occurs around day 21. At day 28, nude mice appeared emaciated, shortness of breath and other symptoms. Tumor in situ filled the entire left chest.

**3:**
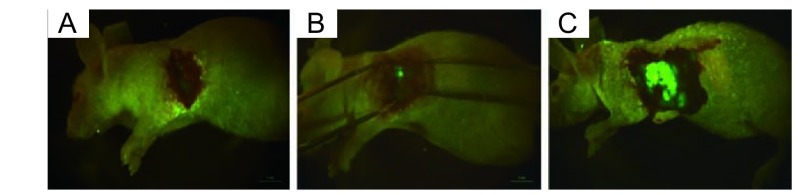
裸鼠原位肿瘤的活体连续观察。A：第7天；B：第14天；C：第21天。 Continuous observation of orthotopic tumors *in vivo*. A: Day 7; B: Day 14; C: Day 21.

**4:**
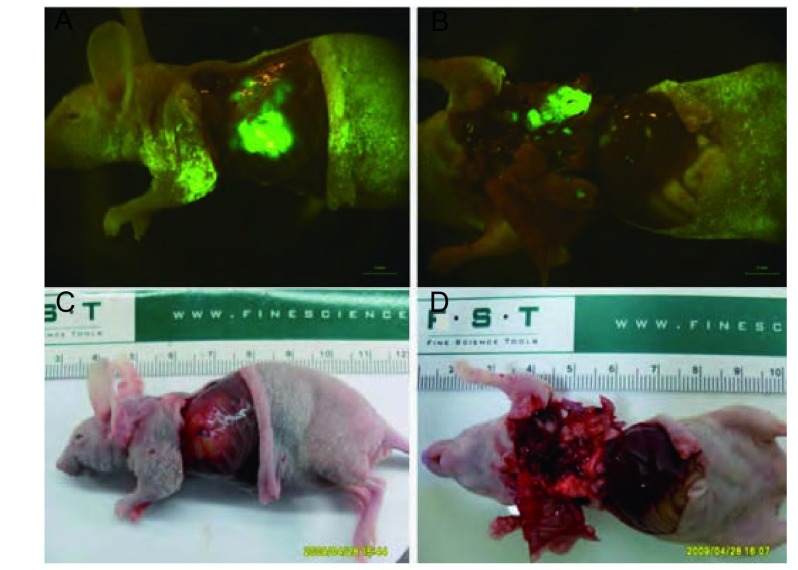
裸鼠自然死亡肿瘤的解剖观察。A、B：荧光成像；C、D：大体照片。整体荧光成像对微小转移灶显像比自然光下大体照片显像更为清晰。 Observation of nude mouse after thoracic anatomy. A, B: Fluorescence imaging; C, D: The general picture. Compared to the general picture, the fluorescence imaging is more clear to detect minor tumours.

### 2.3 裸鼠肺癌原位移植瘤的荧光定量及肿瘤生长曲线的绘制

处死裸鼠后取出原发灶，在荧光体视显微镜下拍照记录并按常规方法测量计算肿瘤体积。经过后期IPP软件处理原位移植瘤荧光面积，对荧光面积和肿瘤体积进行相关性分析。结果表明原位肿瘤荧光面积与肿瘤体积具有相关性（*r*=0.873, *P*=0.001）（图 5）。根据肿瘤面积绘制原位移植瘤生长曲线，可见肿瘤在第2周-第4周生长较快，4周后肿瘤因局限在胸腔内生长，增长速度减慢（图 6）。

**5:**
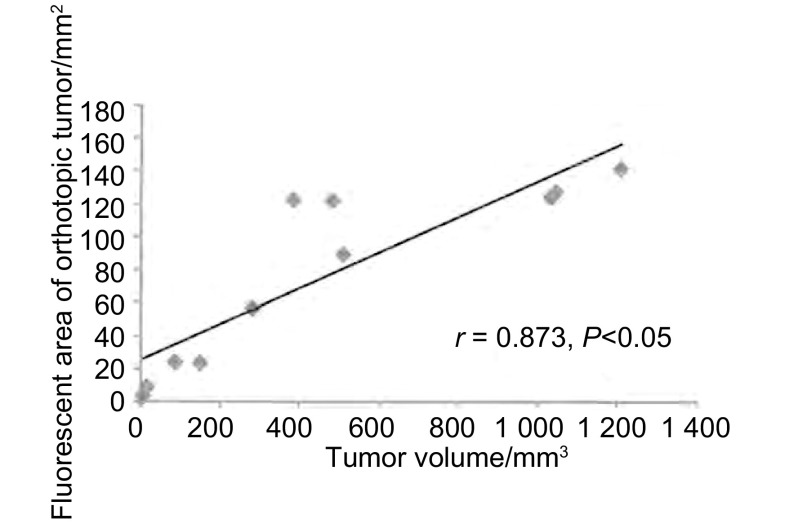
原位肿瘤荧光面积和肿瘤体积之间的相关性分析。原位肿瘤荧光面积和肿瘤体积具有显著相关性（*r*=0.873, *P* < 0.05）。 The correlation analysis between tumor volume and green fluorescent area during tumor proliferation. Significant correlation (*r*=0.873, *P* < 0.05) was observed between tumor volume and green fluorescent area.

**6:**
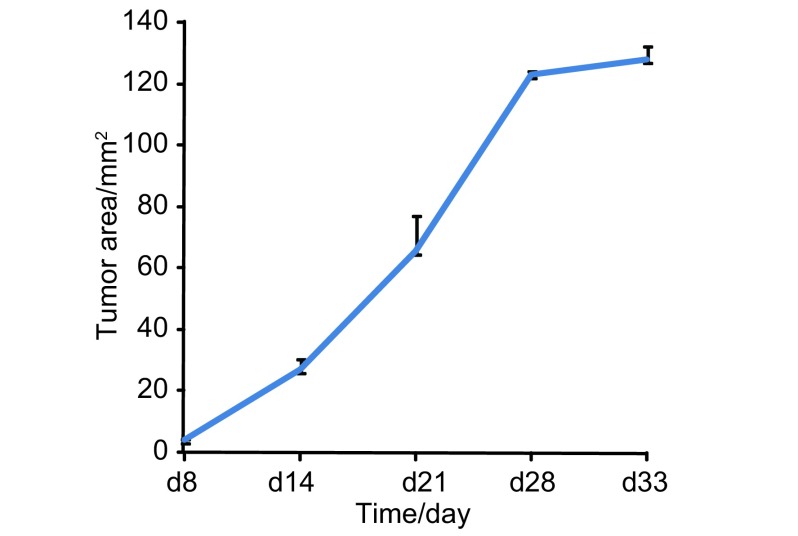
以荧光面积为观察指标为观测指标绘制的生存曲线。第1周-第4周，随着移植时间的增加，表达绿色荧光蛋白的面积逐渐增大，4周后，荧光面积增长速度明显减慢。 The tumor growth of NCI-H460-EGFP cells and dynamic change of fluorescent area. After the establishment of orthotopic model, the area of green fluorescent protein expression of tumor increased gradually during 1 week to 4 weeks. But after 4 weeks, the growth of fluorescent area has slowed significantly.

## 3 讨论

体内移植模型是肿瘤实验研究的主要方法。1969年Rygaard等^[7]^首次成功地在裸小鼠中移植人结肠癌以后，人们开始利用裸小鼠进行肿瘤移植的研究。皮下移植动物模型及转基因动物模型均不能很好地体现临床转移和药物敏感性。1991年Fu等^[8]^创立了外科原位移植方法，它采用的移植物直接来自新鲜外科标本或者移植性人肿瘤细胞或瘤细胞系，通过手术将肿瘤原位移植至裸鼠体内建立肿瘤模型。众多学者通过前列腺癌^[9]^、胰腺癌^[10]^、乳腺癌等原位移植瘤的动物实验模型证实原位移植模型技术是一种巨大的进步，它的转移率和转移模式更接近于临床。

1994年Chalfie等^[11]^首次在大肠埃希菌和线虫中表达GFP，开创了GFP应用研究的先河。近年来，绿色荧光蛋白已成为应用最为广泛的标记性蛋白质之一。*GFP*基因表达的绿色荧光蛋白受到蓝光或紫光照射时可自发地发出绿色荧光，荧光信号强度高，易于被捕获，无需其它底物或辅助因子的协助便可直接通过荧光显微镜或流式细胞仪等进行检测，观察直观方便；同时，还具有稳定性好、耐受性强、对组织或细胞无毒性和易于构建载体等优点。因此，GFP在肿瘤的生长、疗效评价等方面均已获得广泛应用。

活体动物体内光学成像技术将*GFP*基因直接转染至肿瘤细胞，根据荧光蛋白的观测和定量分析直接反映研究细胞的生物学特性。荧光蛋白内在的生色团，在简单的仪器如带有窄带过滤片和激发滤波片的蓝色发光二极管照射下，能发射蓝色激发光，激发荧光蛋白发光。观察者通过合适的看片灯和约490 nm波长的导光纤维光源，可直接肉眼观察大肿块的荧光显影。对于微小肿瘤转移灶，可以通过荧光解剖显微镜来观察。可调过滤器可将皮肤自发荧光的干扰降至最低，排除宿主自身荧光的干扰。活体成像技术在肺癌方面也有大量深入的研究，前期的研究主要集中在肿瘤转移规律方面。Yang等^[12]^将GFP标记的肺癌细胞注射入裸鼠的尾静脉，利用整体成像系统观察了广泛骨转移的过程。Kondo等^[13]^将GFP标记的6种肺癌细胞株原位移植于重症联合免疫缺陷小鼠，观察肿瘤的转移途径及转移部位，3株出现淋巴转移，3株出现血行转移，其中A549株出现多个部位转移，Mat株出现单个部位转移。

本实验通过逆转录病毒介导的基因转移，将编码*EGFP*的基因转入NCI-H460细胞系，通过G418筛选和有限稀释法，获得一株细胞生物学行为无明显改变的、稳定表达绿色荧光的肺癌细胞株NCI-H460-EGFP，并使用外科原位移植技术建立裸鼠原位移植模型，模拟了人肺癌的生长和转移条件。术后第4周-第5周，裸鼠出现恶病质的表现：消瘦、食欲下降、弓背、呼吸短促。处死后解剖发现肺部肿瘤生长，并发生了广泛的转移。但本实验未观察到骨骼、头颅等转移病灶，可能因为原位移植后肺部原发肿瘤生长，未有远处转移时裸鼠即因肺部病变而死亡。

原位移植法建立的模型为体内深部组织生长，肿瘤生长和播散的测量和评价较为困难。常规采用每隔几天处死一定数量裸鼠后行病理分析，以检测肿瘤的生长和转移。活体荧光成像系统无需处死裸鼠即可实现对浅表肿瘤的生长、体内的播散及远处转移的体外实时监测。2003年Katz等^[14]^建立表达红色荧光蛋白的胰腺原位裸鼠模型，分别获取活体测得肿瘤荧光面积和经典方法测得的肿瘤体积，证明两者密切相关。成像系统通过荧光面积客观描述肿瘤体积的生长，所得数据与肿瘤体积相对应。而复旦大学肝癌研究所建立的肝癌原位移植模型分析荧光积分光密度（integrated optical density, IOD）和肿瘤体积之间的相关关系，认为荧光IOD值更能评估肿瘤体积^[15]^。IOD值，又称积分吸光度，为所测结构范围内各像素光密度值之和，表达不仅与体积相关，还与功能相关。肿瘤坏死部分不分泌荧光蛋白，故IOD值实质上是有功能的活细胞光密度的总和。肺癌原位移植为深部肿瘤，拍照时可因为光线、角度等不同，同一肿瘤而得出不同的荧光IOD值，造成偏差。而荧光面积计算方便，本研究的相关性检验分析结果显示荧光面积和体积存在较强的相关性。所以本研究采用荧光面积代替肿瘤体积绘制肿瘤生长曲线来分析肿瘤生长和转移情况。因此，肺癌原位移植模型结合了原位移植和绿色荧光蛋白的优点，可在同一只裸鼠身上达到连续观察的效果。

对于肺癌等体内深部肿瘤，荧光活体成像在肺癌原位观察仍存在一定的问题：EGFP的发射波长为520 nm，易被周围组织散射，所以在成像深度方面尚有一定缺陷。肺部肿瘤GFP激发出的荧光不能穿透皮肤和胸壁，必须暴露成像，打开皮窗，且无法观察内脏器官的远处转移^[16]^。随着研究人员对荧光蛋白的深入探讨，发现发射光谱在近红外波段的荧光蛋白更适合深部动物组织成像。红色荧光蛋白产生的荧光显像将更敏感，周围组织的吸收和散射减少。目前已发现的最亮的荧光蛋白是一种叫Katushka的红色荧光蛋白，发射波长超过620 nm，具有快速成熟、高pH稳定性和光稳定性的特点，将来可能会使肺部肿瘤活体动物的非侵入全身成像成为现实^[17, 18]^。

综上所述，本实验利用SOI法构建的表达绿色荧光蛋白的肺癌裸鼠原位模型，通过观察绿色荧光蛋白能够客观评价NCI-H460-EGFP细胞在裸小鼠体内的生长情况，从而发现肉眼无法发现的微小转移灶，在探索肺癌的生长、转移等机制的研究中有重要的价值。同时本研究还证实了在该模型中可以通过测量荧光面积推测肿瘤体积，从而定量分析肿瘤在体内的生长情况，为药物治疗的临床前研究提供新的实验工具。
